# Predicting protein network topology clusters from chemical structure using deep learning

**DOI:** 10.1186/s13321-022-00622-7

**Published:** 2022-07-15

**Authors:** Akshai P. Sreenivasan, Philip J Harrison, Wesley Schaal, Damian J. Matuszewski, Kim Kultima, Ola Spjuth

**Affiliations:** 1 Department of Pharmaceutical Biosciences, Uppsala University, Box 591, 75124 Uppsala, Sweden; 2 Centre for Image Analysis, Department of Information Technology, Uppsala University, Uppsala, Sweden; 3 Department of Medical Sciences, Uppsala University, Uppsala, Sweden

**Keywords:** Deep learning, Neural networks, Drug discovery, Network topology, Machine learning

## Abstract

Comparing chemical structures to infer protein targets and functions is a common approach, but basing comparisons on chemical similarity alone can be misleading. Here we present a methodology for predicting target protein clusters using deep neural networks. The model is trained on clusters of compounds based on similarities calculated from combined compound-protein and protein-protein interaction data using a network topology approach. We compare several deep learning architectures including both convolutional and recurrent neural networks. The best performing method, the recurrent neural network architecture MolPMoFiT, achieved an F1 score approaching 0.9 on a held-out test set of 8907 compounds. In addition, in-depth analysis on a set of eleven well-studied chemical compounds with known functions showed that predictions were justifiable for all but one of the chemicals. Four of the compounds, similar in their molecular structure but with dissimilarities in their function, revealed advantages of our method compared to using chemical similarity.

**Supplementary Information:**

The online version contains supplementary material available at 10.1186/s13321-022-00622-7.

## Introduction

In the early days of drug discovery and development, researchers believed that chemically similar molecules would have similar biological functions. Although this stands true for many molecules, there are numerous examples where this is not the case. Considerable differences in biological function can even be seen in chemicals with only bioisosteric replacements of atoms/groups and optical enantiomers [1]. When chemicals differs by a single chemical transformation such as the substitution of a hydrogen atom by a chlorine one, they are referred to as matched molecular pairs (MMP) [2]. Further, molecules that are very similar but have large changes in activity have led to the concept of ’activity cliffs’ [3, 4] as they diverge from the underlying assumption of a smooth activity landscape where similar structure implies similar function. Hence, the traditional methods used to ascertain biological similarity, such as comparing the 2D structural fingerprints between molecules with known and unknown biological activities [5], cannot be fully relied upon.

Inferring functions by predicting one or more protein targets for a compound using machine learning algorithms is a widely used methodology. Examples include TargetNet [6] and the method by Lampa et al. [7] which both are based on a battery of models trained on experimental interaction data between compounds and target proteins, extracted from the ChEMBL database. Other approaches include the prediction of metabolic pathways [8–11] and the prediction of the biological process [12] through the use of a variety of methods including likelihood calculation, nearest neighbor algorithms, and mathematical models. Two of these studies [8, 11] were limited only to the chemicals present in the databases such that extrapolation to unknown compounds was not possible.

Another means of comparing chemical structures is to define a measure of biological similarity [13], for example, Muthas et al. predicted safety concerns based on pharmacological similarity using experimental data from biological assays [14]. In another method, known as QuantMap [15], quantitative molecular network topology analysis was used to assess the biological similarity between chemicals. The original implementation of this method was somewhat cumbersome, requiring manual user input, but was streamlined and automated in a subsequent publication [16].

The previously mentioned methodologies for predicting targets and functions based on chemical structure used various machine learning methodologies such as random forests and support vector machines [6, 7]. Recently, deep learning has emerged as an alternative methodology that has shown impressive performance on a variety of classification and regression tasks in cheminformatics [17–19]. Typical input features for quantitative structure-activity relationships (QSAR) consists of molecular fingerprints, Simplified Molecular Input Line Entry System (SMILES) strings [20], fine-tuned molecular descriptors or a combination of the above. Compared to traditional machine learning methods such as random forests and support vector machines, deep learning possesses the advantage of extracting meaningful information required to represent the data directly from the raw input. In QSAR, this means that the chemical structures, such as in the form of SMILES strings, can be used directly rather than molecular descriptors derived from these strings. Deep learning has in some cases been shown to outperform other machine learning methods at deducing complex relationships in the data [17–19, 21].

In this manuscript, we introduce a new method for predicting protein clusters, constructed on the basis of a similarity measure from network topology analysis of compound-protein and protein-protein interaction data. Our method expands the work of Edberg et al. [15] and Schaal et al. [16] by constructing a supervised learning model enabling predictions for novel compounds (i.e. compounds for which there is no information, or limited and poor quality information, in the STITCH and STRING databases). In our method chemical-protein interactions from STITCH [22] and protein-protein interactions from STRING [23] are used to generate a network of interactions for each chemical. The networks are then clustered into groups with biologically similar effects. Subsequently, machine learning models are compared for assigning chemicals to these clusters. For the modeling, we explored several deep learning approaches and validated the final selected model both quantitatively and qualitatively on a set of well-known compounds.

## Methods

### Data and clustering

**Fig. 1:**
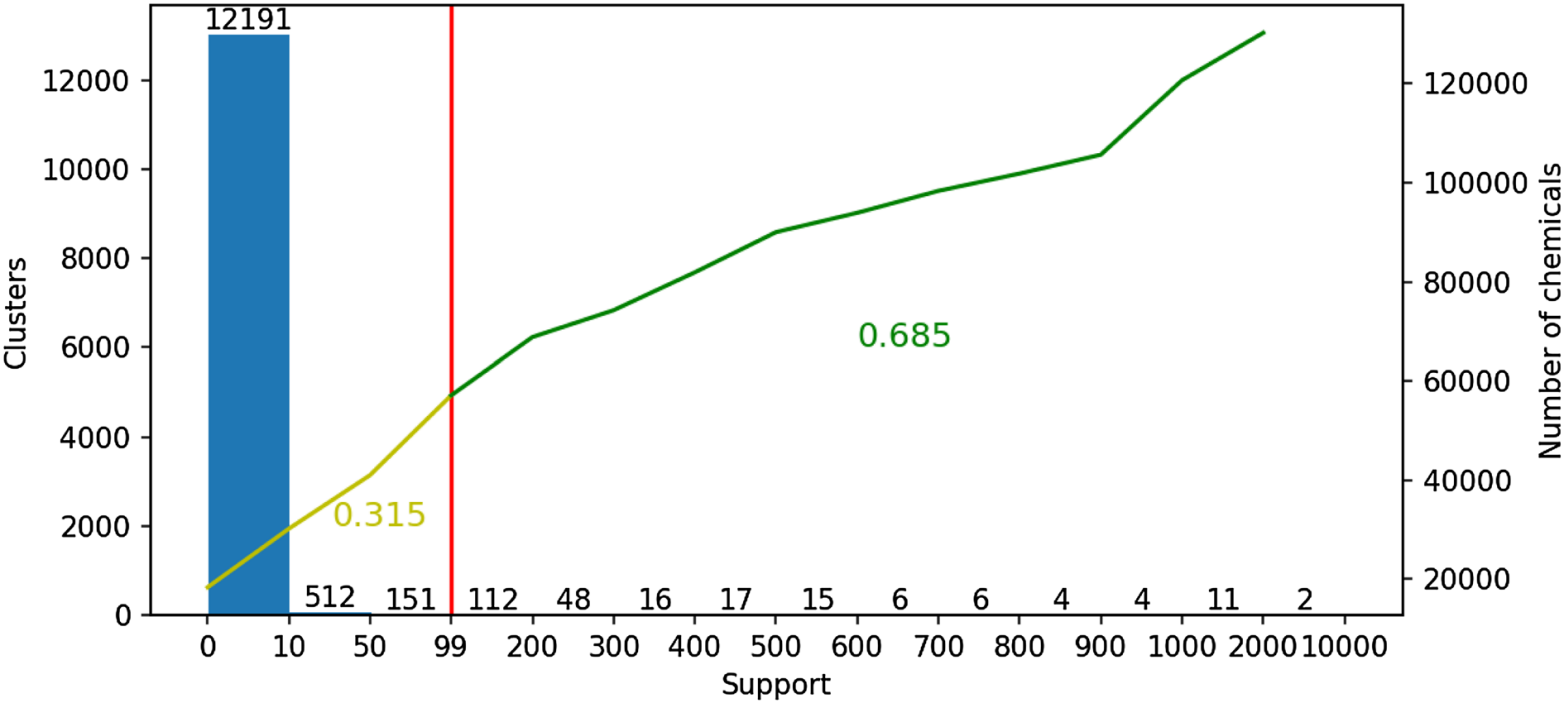
Histogram showing clusters based on their support (using a distance threshold of 0.005). The bars show the number of clusters within the range of support. The yellow and green lines represent the discarded and selected chemicals (support greater than 100), respectively

Interaction data was extracted from the STITCH and STRING databases for the species homo sapiens, where STITCH contains chemical-protein interactions and STRING protein-protein interactions. We used the same versions of the databases as used by Schaal et al. [16] to maintain consistency with these previous experiments. Applying network topology analysis to this data using QuantMap, with the default values for data inclusion (based on a quality criterion) a distance matrix describing the relatedness between 130259 chemicals was obtained; this can be interpreted as a biological distance in the network topology space spanned by the interaction data. In order to produce functional clusters, we used hierarchical clustering [24] on the distance matrix for a range of distance thresholds (0.001, 0.005, 0.01, 0.05 and 0.1). For the chemicals in each clusters, a set of proteins interacting with these chemicals were obtained from STITCH database and assigned as the proteins belonging to the cluster. Clusters with support greater than 100 (i.e., those with more than 100 chemicals) were retained and labeled for further pre-processing (see Table 1). As can be seen in Fig 1, there were many small clusters with only 1-10 compounds that were filtered out.

For all molecules in all resulting clusters, SMILES strings were generated from their 3D representations using RDKIT [25]. A SMILES string is a 2D representation, containing atoms and their arrangement in a molecule. If the 3D representations did not exist, isomeric SMILES or canonical SMILES were obtained from PubChem [26, 27].

**Table 1:** Clustering results of the data using different distance thresholds. The results are shown for both the entire dataset and for clusters with support above 100

	Distance	0.001	0.005	0.01	0.05	0.1
Entire dataset	Chemicals	130259	130259	130259	130259	130259
Entire dataset	Clusters	14057	13095	12162	8293	5680
Support greater than 100	Chemicals	86443	89216	93010	105856	112739
Support greater than 100	Clusters	249	241	231	153	112

### Pre-processing of chemical structures

In our work we used different deep learning approaches to predict functional clusters based on chemical structures. These approaches require different forms of transformations of the molecular data (the SMILES strings).

#### Data pre-processing for deep neural networks

For the Deep Neural Networks (DNNs) the SMILES strings were converted to bit vectors of Morgan fingerprints [28]. These fingerprints are commonly used for molecular comparison, clustering, structure-activity modeling, and deep learning [5, 29]. Morgan fingerprints are a re-implementation of the Extended Connectivity Fingerprints (ECFP) [30], a class of topological fingerprints representing molecular structures and particular substructures. They are computationally inexpensive to calculate and are unique to a molecule. In this work, a fingerprint with a radius of 2 and a bit vector of size 1024 was used to represent the molecules. As each Morgan fingerprint is unique for a given molecule, data augmentation cannot be applied.

#### Data pre-processing for convolutional neural networks

**Fig. 2:**
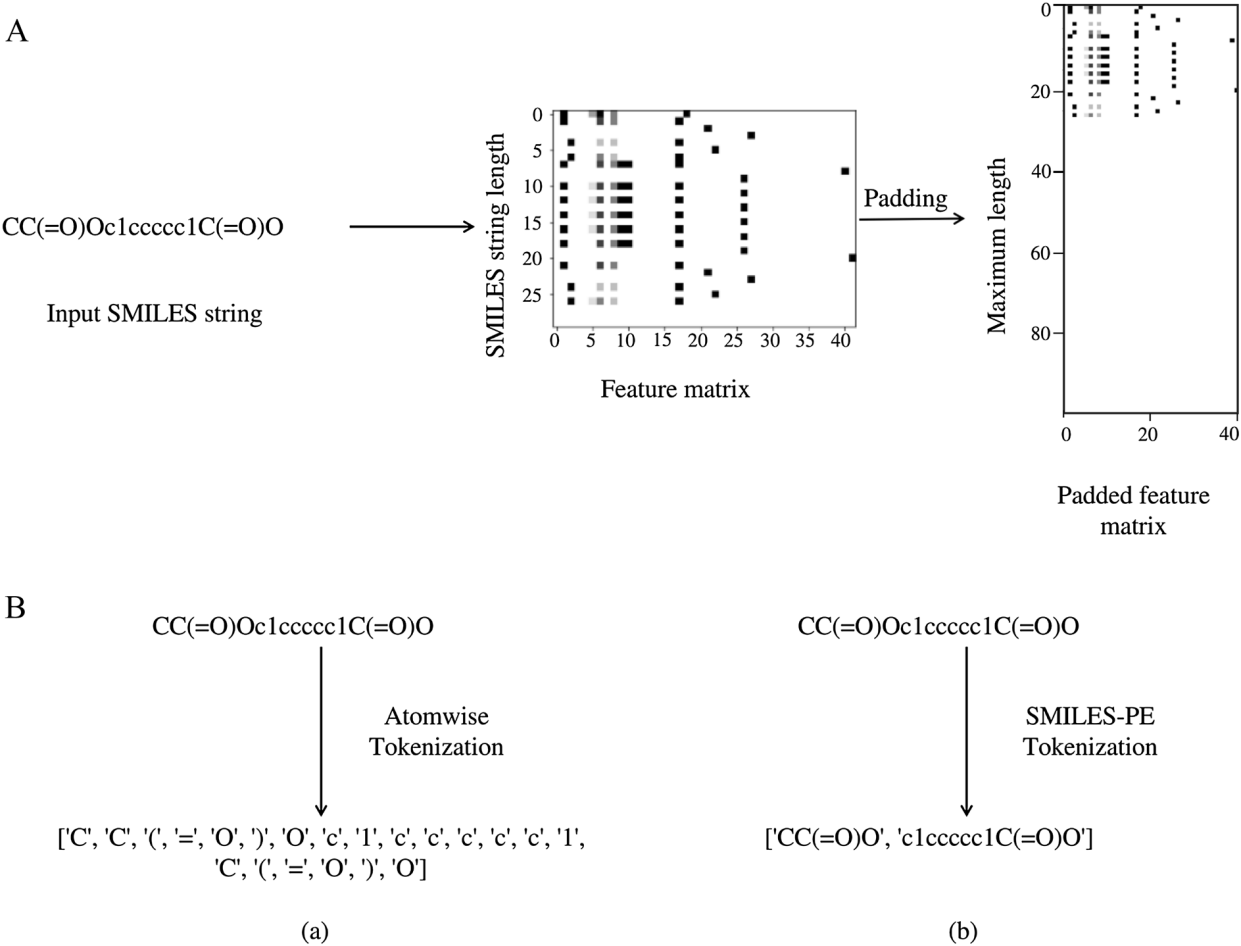
Data pre-processing for CNN and RNN architectures. **A** An example of SMILES string conversion to a matrix of dimension 42 X (length of SMILES string). This matrix is padded along the y-axis up to a defined maximum length. **B** (a) Atomwise and (b) SMILES-PE tokenization for the compound aspirin

The data was prepared for Convolutional Neural Networks (CNNs) by converting the SMILES strings to feature matrices using the method proposed by Hirohara et al. [31] (Fig 2A). Data augmentation cannot be used either for this representation as the SMILES strings had to be converted to their canonical form before being transformed to feature matrices.

#### Data pre-processing for Recurrent Neural Networks

For Recurrent Neural Networks (RNNs) the input SMILES strings were converted to tokens using the methods of Li & Fourches [32]. Both atom-wise and SMILES-PE tokenizations were explored as inputs to the RNNs (Fig 2B). These tokenized strings are inputted to an embedding matrix, which discerns the relationship between the input tokens. These embeddings are then passed on to the RNN cells. Both the embedding matrix and the weights of the RNN cells are updated during model training.

### Data augmentation

Data augmentation is a popular technique used in deep learning to compensate for lack of variation in the training data and often improves the model’s ability to generalize to unseen data [33]. The training data can be augmented in various ways depending on the input data type and the application [34]. For SMILES string data, the atoms and groups can be reordered without losing the structural integrity of the molecule. Hence a single molecule can be represented in multiple ways using SMILES annotation. For instance, the aspirin molecule can be augmented (see Additional file 1: Fig S1) to 144 different (yet equivalent) SMILES representation. The maximum number of augmentations depends on the complexity and size of the molecule, and how it can be reordered. This method of SMILES augmentation has shown to improve the performance of deep learning models [33]. In our work, data augmentation was only applicable for the RNN modeling. To compensate for cluster imbalance, class-wise augmentation of the data was implemented by augmenting the clusters with fewer compounds a larger number of times than those with many compounds.

### Deep learning architectures

All models were trained with categorical cross-entropy loss and Adam optimizers [35]. The hyperparameters, including the learning rate, number of epochs, batch size, network size, dropout rate [36], and activation functions [37] were tuned to obtain the optimal performance from each model.

#### Deep neural network

A fully connected Deep Neural Network (DNN) architecture consisting of 3 hidden layers with 4096, 4096, and 1024 neurons, respectively, was used. Each layer was followed by a ReLU activation function and dropout with a rate of 0.4. Alternative choices for the number of hidden layers and neurons per layer was investigated (results not shown), but this 3 layer neural network performed the best.

#### Convolutional neural network

**Fig. 3:**
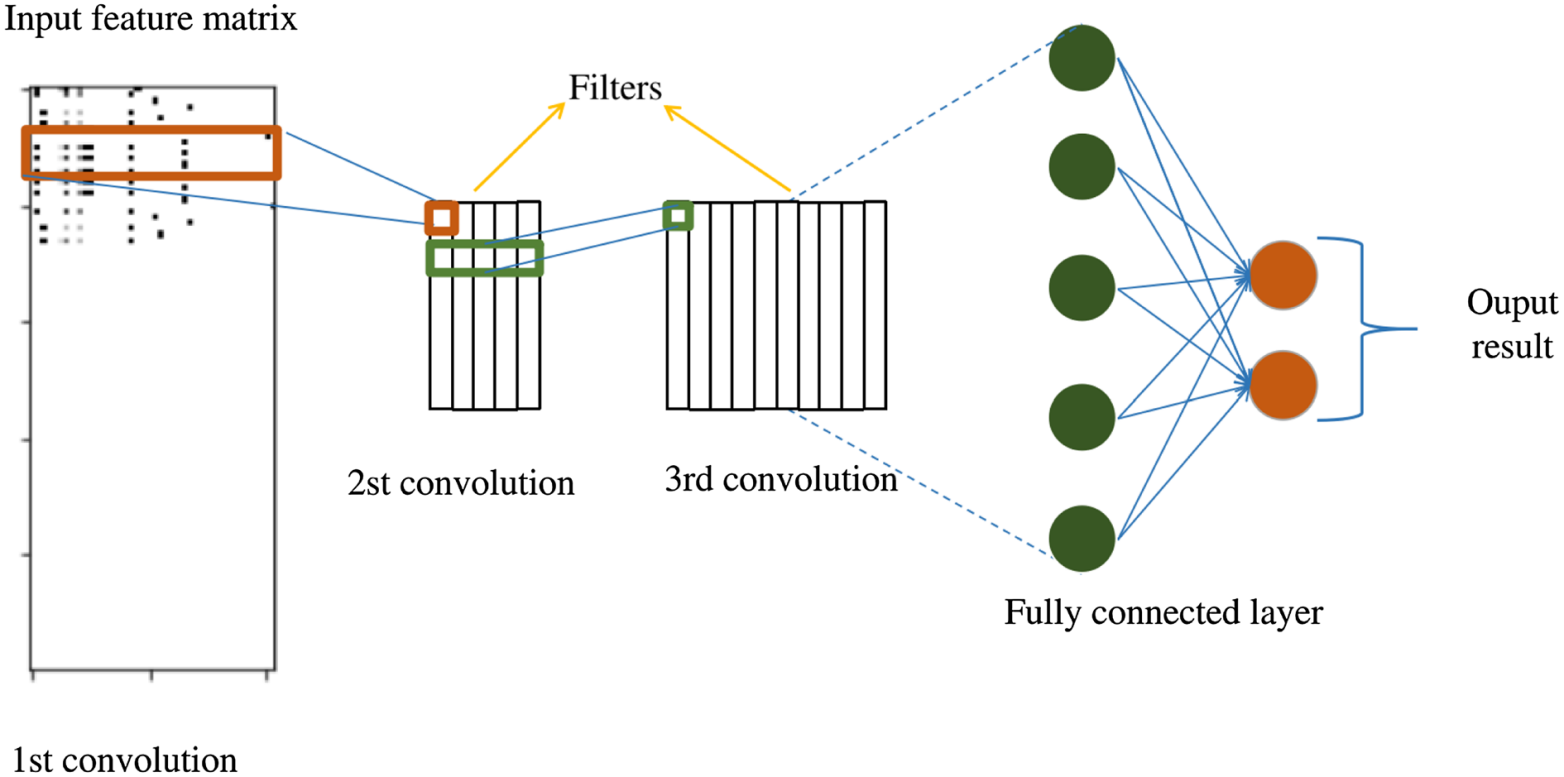
Architecture of the CNN used. The given network takes the feature matrix as input and has three convolutional layers followed by a fully connected layer prior to the final classification layer

Convolutional Neural Networks (CNNs) are the network architecture of choice for many computer vision applications [38]. They can also be applied to data stored in a matrix form. The CNN architecture used in this study was adopted from the network proposed by Hirohara et al. [31] (Fig 3). The network parameters were kept at their default values except for the sequence length cutoff parameter. In our work, this parameter was increased to accommodate the length of the longest compound sequence.

#### Recurrent neural network

**Fig. 4:**
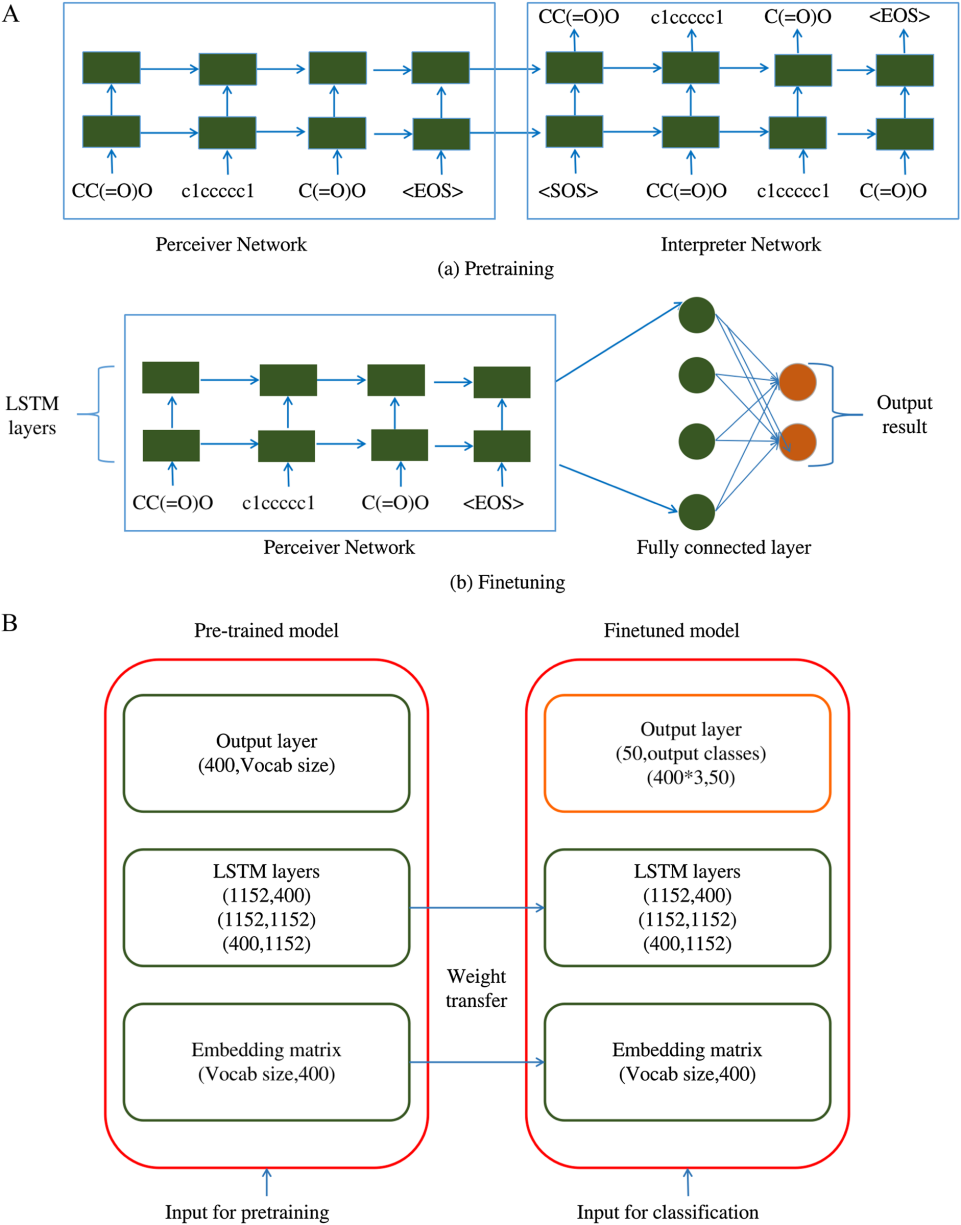
Two different RNN architectures used: **A** (a) Seq2seq architecture with both perceiver and interpreter networks. The perceiver network is pre-trained using unlabelled data, to learn patterns and structures present in the data. (b) Finetuning of seq2seq network, where the perceiver network is connected to a fully connected layer for classification. **B** Pre-training and fine-tuning of the MolPMoFiT model. Weights from the pre-trained embedding matrix and three layers of LSTM are transferred and fine-tuned to perform the classification task at hand

Recurrent Neural Network (RNN) architectures are widely used for applications involving temporal or sequential data and can accommodate input data of variable lengths [39]. For QSAR applications, pre-processed SMILES strings (Data pre-processing for Recurrent Neural Networks) can be given as input. In this study, two RNN-based methods were explored:

1. Seq2seq: The Seq2Seq model, adapted from Xu et al. [40], consists of a perceiver and an interpreter network (Fig 4A). The interpreter network is an optional component implemented during the pre-training of the network. In this paper, a perceiver network with 3 layers of LSTMs, each consisting of 256 hidden state units, was used. The interpreter network was omitted as we had a sufficient amount of data available for training.
2. MolPMoFiT: The MolPMoFiT architecture was adapted from Li and Fourches [41]. This architecture is a modification of the Natural Language Processing (NLP) model, ULMFiT, for chemical data in the form of SMILES strings as opposed to text. The MolPMoFiT model required both pre-training and fine-tuning (Fig 4B). The pre-training of the model was accomplished using SMILES from the STITCH database. Three pre-trained models were created using different input data variations: atom-wise tokenization, SmilesPE tokenization, and SmilesPE tokenization with augmentation, respectively.

### Study design

**Fig. 5:**
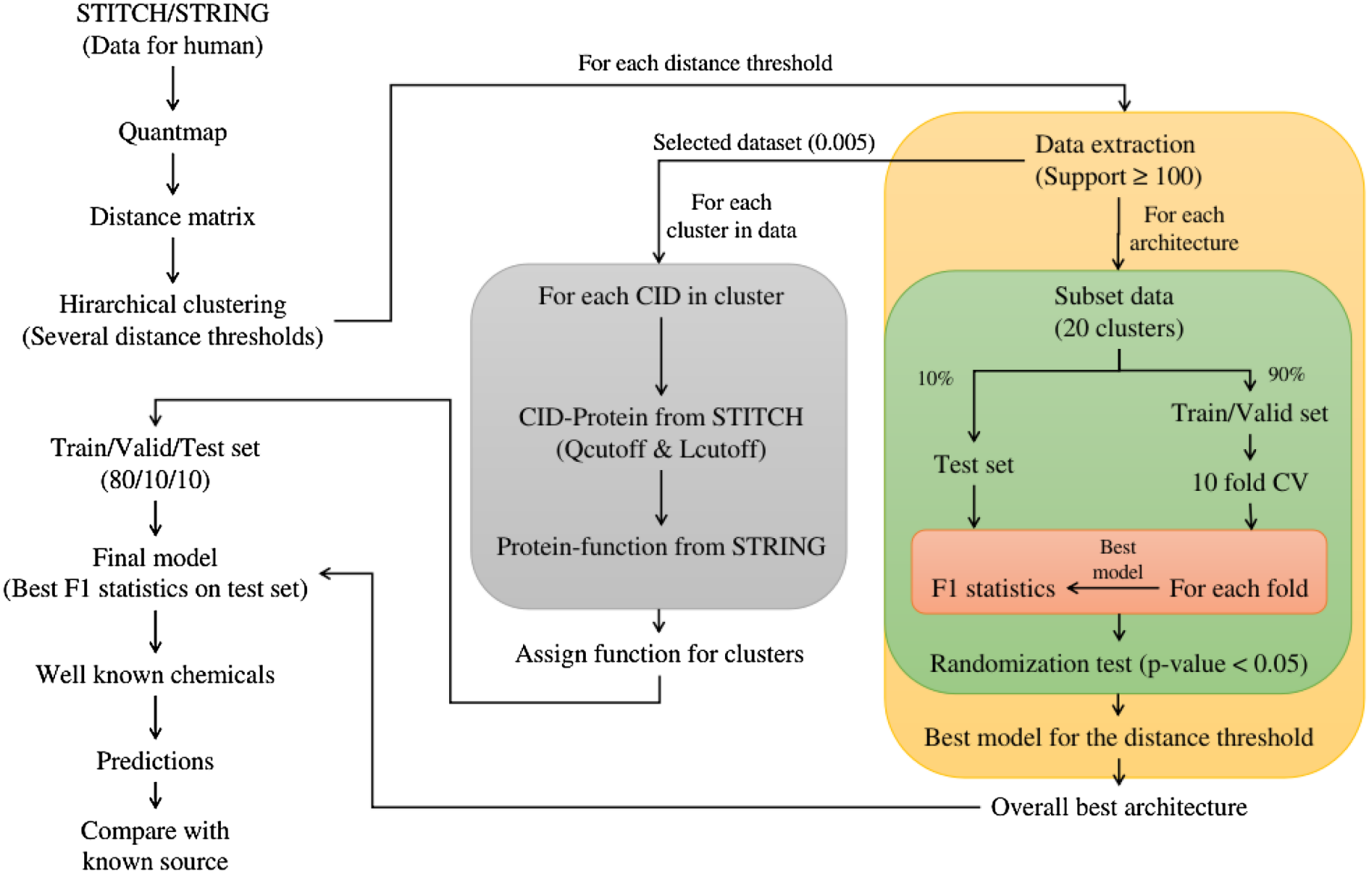
Workflow used in this study. The data was obtained from STITCH and STRING databases and were processed using Quantmap followed by hierarchical clustering using several distance thresholds. For each distance threshold, a subset of 20 clusters was used to evaluate different deep learning architectures. Further, a dataset of interest was selected for training and functional assignment of clusters was carried out. The final trained model was later evaluated using well-known chemicals

The workflow used in this study is given in Fig. 5. For the evaluation of different deep learning architectures and parameters, a subset of 20 clusters was chosen for each dataset corresponding to a specific distance value. Care was taken in this subset selection to include both closely related and distant clusters. From the obtained subsets of data, 10% was held out for test time assessment. The remaining 90% of the data was used in 10-fold cross-validation [42]. For each fold in the cross-validation, test set statistics were calculated using the best performing model on each fold. An overall best architecture was then selected and applied to the entire dataset (all clusters).

When training on all clusters the data was split into 80/10/10 for training, validation, and testing, respectively. Performance on the test set was then evaluated based on the F1 statistic.

To assess the real-world applicability of the model, the cluster assignments for a set of well-known chemicals were investigated using the final model. These chemicals were not included in the data used for training or validation. The known functions of these chemicals were compared to the functions represented in their assigned clusters. To assign properties to a particular cluster, the set of proteins interacting with the chemicals in the clusters were obtained from the STITCH database. The functions for the obtained set of proteins were assigned as the functions of the cluster. Two sets of functional data were generated for the clusters, one using the higher quality cutoff parameter (Qcutoff) and the other one using a lower cutoff parameter (Lcutoff), which includes an increased set of proteins and their functions, but with lower confidence.

## Results

### Architecture comparisons

**Fig. 6:**
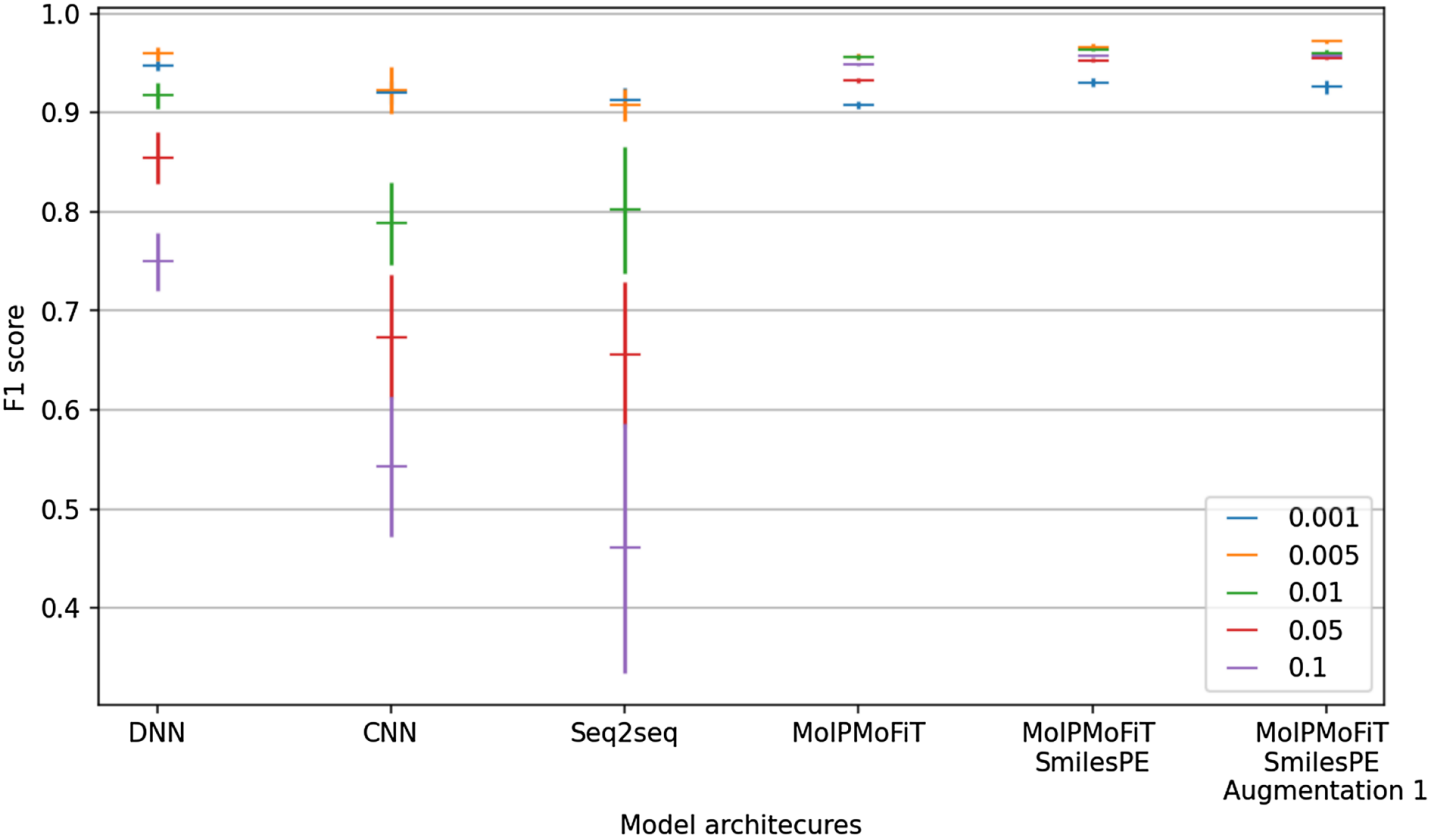
Comparison between different architectures. F1 score means and standard deviations (for ten cross-validation folds) of the deep learning models compared on the five clustering distance thresholds

Cross-validation was carried out for the obtained subsets of data from each distance threshold derived dataset. From the F1 statistics and their standard deviations (Fig. 6), and randomization tests (see Additional file 1: Fig S2), it is evident that MolPMoFiT with atom-wise tokenization outperformed the Seq2Seq model with atom-wise tokenization (randomization test p-values < 0.05 for all distance thresholds apart from 0.001). In light of this generally superior performance of the MolPMoFiT model, the effect of different tokenization methods (atom-wise and SmilePE tokenization, Data pre-processing for Recurrent Neural Networks) was evaluated for this model. Significantly higher performance was achieved by SmilesPE tokenization, compared to atomwise tokenization (p-value <  0.05). Further performance improvements to this model were achieved with the addition of class-wise augmentation (Data augmentation). Comparing the results between SmilesPE and SmilesPE with augmentation, there was a noticeable gain in the performance on the datasets with distance thresholds 0.005, 0.01, and 0.05 (p-value < 0.05) but not for the datasets with thresholds of 0.001 and 0.1. Owing to this heterogeneous performance, both architectures were retained for further assessment.

### Model training and evaluation

For assessment of the best performing model, MolPMoFiT, the clustering obtained for the entire dataset, using an intermediate distance threshold of 0.005, was used. This resulted in 241 clusters of 89216 chemicals (Table 1). The data was split into training (80%), validation (10%) and test (10%) sets, corresponding to 72272, 8037 and 8907 compounds, respectively.

The training of the final MolPMoFiT model with SmilesPE tokenization, was carried out in the following ways: (1) without augmentation (for comparison), (2) with one class-wise augmentation, and (3) with five class-wise augmentations and obtained test set F1 scores of 0.831, 0.873 and 0.887, respectively. Based on the F1 scores, there is a clear performance improvement when using data augmentation. Therefore the MolPMoFiT model utilizing SmilesPE tokenization with five class-wise augmentations was selected for further evaluation.

### Application to well known chemicals

**Fig. 7:**
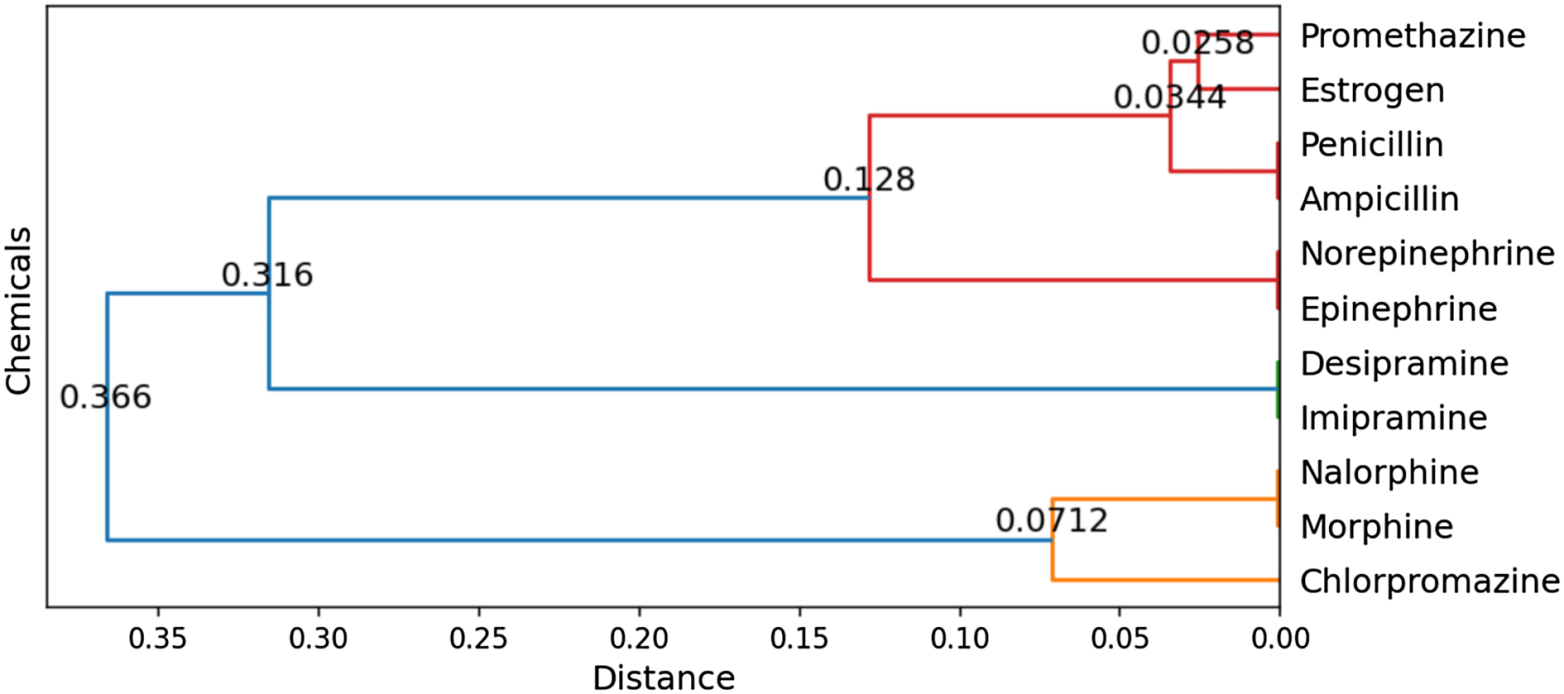
Predicted distances between the eleven chemicals selected for in-depth analysis

The functions for a set of chosen chemicals excluded from the model training (morphine, nalorphine, estrogen, penicillin, ampicillin, nor-epinephrine and epinephrine, imipramine, desipramine, chlorpromazine, and promethazine) were predicted using the final model and the distances between these chemicals (i.e, distances between the predicted clusters for the chemicals) were determined using cluster distances obtained initially from hierarchical clustering (Fig. 7). The predicted clusters were assigned function using both the Qcutoff and Lcutoff (see Table 2), where the top five proteins are shown for each cluster assignment. These predicted functions were compared against the DrugBank annotations for the chosen chemicals [43].

**Table 2:** Comparison of the functional groups predicted for the eleven evaluation chemicals against their DrugBank annotations. Functions annotated in italics or bold are exclusive for the compound in the same font type

Compound	Qcutoff	Lcutoff	DrugBank annotation
Morphine	Kappa-type opioid receptor	Kappa-type, Mu-type and Delta-type opioid receptor, Cytochrome P450 2D6, Proenkephalin-B	Kappa-type, Mu-type and Delta-type opioid receptor, Lymphocyte antigen 96
Nalorphine			Not found
**Ampicillin**, *Penicillin*	Solute carrier family 15 member 1 and 2	Solute carrier family 15 member 1 and 2, Angiotensin- converting enzyme, Protein polybromo-1, Band 3 anion transport protein	Solute carrier *family 22 member 8*, family 15 member 1 and family 15 member 2, **Angiopoietin-1 receptor**
**Epinephrine**, *Norepinephrine*	Beta-2 adrenergic receptor	Beta-1, 2, and 3 adrenergic receptor, Extracellular calcium-sensing receptor, ER membrane protein complex subunit 6	Alpha-1A, 1B, 1D, 2A, 2B and *2C* adrenergic receptor, Beta-1, 2 and **3** adrenergic receptor, *Phenylalanine-4-hydroxylase , Synaptic vesicular amine transporter, Chromaffin granule amine transporter*,**Tumor necrosis factor**
Estrogen	Carbonic anhydrase 9, 2, 1, 12, 14, 7, 5A, 5B and 13	Carbonic anhydrase 9, 2, 1, 12, 14, 7, 5A, 5B and 13	Estrogen receptor alpha and beta, Nuclear receptor subfamily 1 group I member 2, Neuronal acetylcholine receptor subunit alpha-4, G-protein coupled estrogen receptor 1, ATP synthase subunit a, Beclin-1
**Imipramine**, *Desipramine*	5-hydroxytryptamine receptor 2A; G-protein coupled receptor for 5-hydroxytryptamine (serotonin)	5-hydroxytryptamine receptor 2A, 2C, Potassium voltage-gated channel subfamily H member 2, D(2) dopamine receptor, Sodium-dependent serotonin transporter	Sodium-dependent noradrenaline, serotonin and **dopamine** transporter , Histamine H1 receptor, Muscarinic acetylcholine receptor M1, M2, M3, M4 and M5, 5-hydroxytryptamine receptor 1A, 2A, 2C, **7 and 6, D(1)** and D2 dopamine receptor, **Potassium voltage-gated channel subfamily H member 1 and 2, D member 2 and 3 , Alpha-1A, 1D and 1B adrenergic receptor , Alpha-1-acid glycoprotein 2**, *Sphingomyelin phosphodiesterase, Alpha-1,2,Beta-1,2 adrenergic receptor*
Chlorpromazine	Dopamine receptor D4	D4, D(2), D(3) dopamine receptor, 5-hydroxytryptamine receptor 1A, 5-hydroxytryptamine receptor 2A	D(1), D1, D2, D3, D4, and D5 dopamine receptor, 5-hydroxytryptamine receptor 1A, 2A, 2C, 2, 6, and 7, Alpha-1A and 1B adrenergic receptor, Histamine H1 and H4 receptor, Potassium voltage-gated channel subfamily H member 2, Alpha-1 and 2 adrenergic receptors, M1 and M3 muscarinic acetylcholine receptor, Sphingomyelin phosphodiesterase, Calmodulin, Alpha1-acid glycoprotein
Promethazine	Cholinesterase, Testis, prostate and placenta expressed	Cholinesterase, Acetylcholinesterase, Acetylcholinesterase collagenic tail peptide, Amyloid-beta A4 protein, Potassium voltage-gated channel subfamily H member 1	Histamine H1 receptor, Histamine H2 receptor, Dopamine D2 receptor, Muscarinic acetylcholine receptor M1, M2, M3, M4 and M5, Alpha adrenergic receptor, Calmodulin, P2 Purinoceptors, Voltage-gated sodium channel alpha subunit, Voltage-gated Potassium Channels

For the chemical nalorphine, there is currently no DrugBank annotation, but it is known that it acts as an antagonist while morphine acts as an agonist [1]. Since both of these chemicals act on the same set of proteins, but with opposite interactions, it is understandable that they were assigned to the same cluster. A similar result can be seen for norepinephrine and epinephrine, both of which are $\alpha$-adrenegic agonists [1]. This also holds true for penicillin and ampicillin, where both have been experimentally proven to act on the same set of proteins.

Concerning the chemicals with similar structures and different functions, our model predicted that chlorpromazine act on dopamine receptors, promethazine on cholinesterases and ion channels, while imipramine and desipramine act on neurotransmitters. This corresponds well with their evidence from article [1] and DrugBank annotation (Table 2). Hence, despite their structural similarity, as can be seen in Additional file 1: Fig S3, these chemicals were assigned to appropriate functional clusters.

However, for the chemical estrogen, the predicted functions were not in line with those of DrugBank. Nevertheless, if we look at the second and third best clusters for this chemical, based on the sorted softmax probabilities prior to the prediction made for this model, we see functions for this chemical that are more in line with those of DrugBank (Additional file 1: Table S1). The sorted softmax probabilities for these top three clusters were 0.9233, 0.0397, and 0.0202. Although the probability for the top cluster is relatively high in this case, it is known that softmax probabilities are not well calibrated and tend to produce overconfident predictions [44, 45].

## Discussion

A tool that can predict the biological activities caused by chemicals and thus aid in drug discovery, developing generic drugs, and understanding the cascade of induced metabolic activities, is of high demand and interest. In this study we developed such a tool, a tool capable of predicting the proteins interacting with a chemical of interest. This tool has potential for lead identification and drug discovery, and it could also be applied in toxicology studies, shedding light upon possible side effects caused by the chemicals.

Several previous studies have explored the prediction of metabolic pathways [8–11], but without accounting for the cascade of interactions caused by them. Some of these methods were also limited to features such as functional groups, size of the molecules, and descriptors, thus failing to represent a large set of the chemical space. A similar study to ours, using chemical-protein and protein-protein interaction data from STITCH and STRING, was undertaken by Gao et al. [11]. However, their method was limited to metabolic pathway prediction and lacked the ability to make predictions for new compounds. This prediction hurdle was overcome by the Prediction of Biological Activity Spectra for Substances (PASS) algorithm [12] and iFragMent [9] by allowing user-generated input chemical structure to be processed. Even though these methods provide useful knowledge about the possible activity of the molecule, they do not identify the possible interactions leading to this activity, as we do with our method.

In the model comparison part of our study, the best performing model was the RNN model MolPMoFiT, supplemented with an improved tokenization method and data augmentation. The improved performance of the MolPMoFiT model compared to the other RNN model (seq2seq model), is due to the two major features present in the MolPMoFiT model, dropconnect and ASGD (Averaged Stochastic Gradient Descent), along with regularization techniques introduced in the model. Furthermore, AWD-LSTM (the underlying model of MOLPMOFIT) has shown to outperform the other RNN based models for language processing [46].

The predictions of this model were explored for a set of well-studied chemicals, that were not used during the model development. The predicted biological functions were compared to those in DrugBank and elsewhere in the research literature for both high and low confidence data. The functional predictions were reasonable for all but one of the chemicals, estrogen. This could be due to an incorrect classification made by the model or due to unknown interactions of estrogen. As estrogen is such a well-known chemical the latter seems unlikely. It is also possible that the ideal cluster for estrogen had been removed from the dataset. Several small clusters (those with less than 100 compounds) had been removed prior to model training. This was a necessary step as the models required sufficient information for each cluster to make predictions.

Even though the model gave reasonable predictions for most of the molecules, estrogen appeared to be an outlier. This can be explained in two ways: (i) there was not enough evidence in the literature to prove the interaction of estrogen with the receptors in the cluster (ii) the model made an incorrect classification. In a general sense, cases of the former type could aid in future research for identify novel interactions by chemicals, or to expose overlooked interactions (helpful for understanding the side effects of molecules). Cases of the later type could be addressed by incorporating confidence into the model output, for example Conformal prediction [47]. It is also possible that chemicals may belong to multiple classes, based on the proteins with which they interact. Although a molecule may have its major interaction with proteins in one class it may also have relevant interactions in other classes.

## Conclusion

We present a new method for predicting protein targets, and implicitly biological functions, based on chemical structure using a deep learning model trained on data from network topology analysis. The method has high accuracy for predicting the correct protein target cluster, and produces lists of potential functions that were in accordance with a set of well-known compounds. Our results demonstrates the usefulness of complementing target predictions based on chemical similarity with ’biological’ distances from network topology analysis of compound-protein and protein-protein interaction networks.

## Supplementary Information

**Additional file 1: Fig. S1** SMILES augmentation for the molecule aspirin. **Fig. S2** Randomization test results for all the architectures used for different set of data. **Fig. S3** Structures of the chemicals used for evaluating the model. **Table S1** Functions predicted by the model for estrogen predicted for the second and third ranked clusters (based on the output softmax probabilities).

## Data Availability

The code used for data pre-processing and for the various models explored are available at https://github.com/pharmbio/dl_quantmap.

## References

[1] Kubinyi H (2002) Chemical similarity and biological activities. J Brazil Chem Soc 13. 10.1590/S0103-50532002000600002

[2] Griffen E, Leach AG, Robb GR, Warner DJ (2011) Matched molecular pairs as a medicinal chemistry tool. J Med Chem 54(22):7739–50. 10.1021/jm200452d 10.1021/jm200452d 21936582

[3] Maggiora GM (2006). On outliers and activity cliffs-why qsar often disappoints. J Chem Inf Model.

[4] Guha R, Van Drie JH (2008). Structure–activity landscape index: identifying and quantifying activity cliffs. J Chem Inf Model.

[5] Bajusz D, Rácz A, Héberger K (2015) Why is tanimoto index an appropriate choice for fingerprint-based similarity calculations? J Cheminformat 7. 10.1186/s13321-015-0069-3 10.1186/s13321-015-0069-3 PMC4456712 26052348

[6] Yao Z-J, Dong J, Che Y-J, Zhu M-F, Wen M, Wang N-N, Wang S, Lu A-P, Cao D-S (2016). Targetnet: a web service for predicting potential drug–target interaction profiling via multi-target sar models. J Comput Aided Mol Des.

[7] Lampa S, Alvarsson J, Arvidsson Mc Shane S, Berg A, Ahlberg E, Spjuth O (2018). Predicting off-target binding profiles with confidence using conformal prediction. Front Pharmacol.

[8] Hu L-L, Chen C, Huang T, Cai Y-D, Chou K-C (2011). Predicting biological functions of compounds based on chemical–chemical interactions. PLoS ONE.

[9] Lopez-Ibañez J, Pazos F, Chagoyen M (2021) Predicting biological pathways of chemical compounds with a profile-inspired approach. BMC Bioinformat 22. 10.1186/s12859-021-04252-y 10.1186/s12859-021-04252-y PMC8199418 34118870

[10] Cai Y-D, Qian Z, Lu L, Feng K, Meng X, Niu B, Zhao G-D, Lu W-C (2008). Prediction of compounds’ biological function (metabolic pathways) based on functional group composition. Mol Divers.

[11] Gao Y-F, Chen L, Cai Y-D, Feng K-Y, Huang T, Jiang Y (2012). Predicting metabolic pathways of small molecules and enzymes based on interaction information of chemicals and proteins. PLoS ONE.

[12] Stepanchikova AV, Lagunin A, Filimonov DA, Poroikov V (2003). Prediction of biological activity spectra for substances: evaluation on the diverse sets of drug-like structures. Curr Med Chem.

[13] Petrone PM, Simms B, Nigsch F, Lounkine E, Kutchukian P, Cornett A, Deng Z, Davies JW, Jenkins JL, Glick M (2012). Rethinking molecular similarity: comparing compounds on the basis of biological activity. ACS Chem Biol.

[14] Muthas D, Boyer S (2013). Exploiting pharmacological similarity to identify safety concerns-listen to what the data tells you. Mol Inf.

[15] Edberg A, Soeria-Atmadja D, Laurila J, Johansson F, Gustafsson M, Hammerling U (2012). Assessing relative bioactivity of chemical substances using quantitative molecular network topology analysis. J Chem Inf Model.

[16] Schaal W, Hammerling U, Gustafsson MG, Spjuth O (2013). Automated QuantMap for rapid quantitative molecular network topology analysis. Bioinformatics.

[17] Goh GB, Hodas NO, Vishnu A (2017). Deep learning for computational chemistry. J Comput Chem.

[18] Ma J, Sheridan R, Liaw A, Dahl G, Svetnik V (2015). Deep neural nets as a method for quantitative structure–activity relationships. J Chem Inf Model.

[19] Dahl GE, Jaitly N, Salakhutdinov R (2014) Multi-task neural networks for qsar predictions. ArXiv **abs/1406.1231**

[20] Weininger D (1988). Smiles, a chemical language and information system. 1. introduction to methodology and encoding rules. J Chem Informat Comput Sci.

[21] Koutsoukas A, Monaghan K, Li X, Huan J (2017) Deep-learning: Investigating deep neural networks hyper-parameters and comparison of performance to shallow methods for modeling bioactivity data. J Cheminformat 9. 10.1186/s13321-017-0226-y 10.1186/s13321-017-0226-y PMC5489441 29086090

[22] Kuhn M, Szklarczyk D, Franceschini A, von Mering C, Jensen L, Bork P (2011). Stitch 3: zooming in on protein–chemical interactions. Nucleic Acids Res.

[23] Franceschini A, Szklarczyk D, Frankild S, Kuhn M, Simonovic M, Roth A, Lin J, Minguez P, Bork P, von Mering C, Jensen LJ (2012). STRING v9.1: protein–protein interaction networks, with increased coverage and integration. Nucleic Acids Res.

[24] Rokach L, Maimon O (2005) Clustering Methods, pp. 321–352

[25] Landrum G (2016) Rdkit: open-source cheminformatics software

[26] Kim S, Thiessen PA, Cheng T, Yu B, Bolton EE (2018). An update on PUG-REST: RESTful interface for programmatic access to PubChem. Nucleic Acids Res.

[27] Kim S, Chen J, Cheng T, Gindulyte A, He J, He S, Li Q, Shoemaker BA, Thiessen PA, Yu B, Zaslavsky L, Zhang J, Boltom EE (2020). PubChem in 2021: new data content and improved web interfaces. Nucleic Acids Res.

[28] Morgan HL (1965). The generation of a unique machine description for chemical structures—a technique developed at chemical abstracts service. J Chem Document.

[29] Myint K-Z, Wang L, Tong Q (2012). Xie X-Q Molecular fingerprint-based artificial neural networks qsar for ligand biological activity predictions. Mol Pharmaceut.

[30] Rogers D, Hahn M (2010). Extended-connectivity fingerprints. J Chem Inf Model.

[31] Hirohara M, Saito Y, Koda Y, Sato K, Sakakibara Y (2018) Convolutional neural network based on smiles representation of compounds for detecting chemical motif. BMC Bioinformat 19. 10.1186/s12859-018-2523-5 10.1186/s12859-018-2523-5 PMC6311897 30598075

[32] Li X, Fourches D (2021). Smiles pair encoding: a data-driven substructure tokenization algorithm for deep learning. J Chem Inform Model.

[33] Bjerrum EJ (2017) SMILES enumeration as data augmentation for neural network modeling of molecules. CoRR. abs/1703.07076

[34] Hussain Z, Gimenez F, Yi D, Rubin D (2018) Differential data augmentation techniques for medical imaging classification tasks. In: AMIA Annual Symposium proceedings. AMIA Symposium 2017, 979–984 PMC5977656 29854165

[35] Kingma DP, Ba J (2017) Adam: a method for stochastic optimization. 1412.6980

[36] Srivastava N, Hinton G, Krizhevsky A, Sutskever I, Salakhutdinov R (2014). Dropout: a simple way to prevent neural networks from overfitting. J Mach Learn Res.

[37] Bio-inspired neurocomputing (2021) Stud Comput Intell. 10.1007/978-981-15-5495-7

[38] Alzubaidi L, Zhang J, Humaidi AJ, Al-Dujaili A, Duan Y, Al-Shamma O, Santamaría J, Fadhel MA, Al-Amidie M, Farhan L (2021). Review of deep learning: concepts, cnn architectures, challenges, applications, future directions. J Big Data.

[39] Yu Y, Si X, Hu C, Zhang J (2019). A review of recurrent neural networks: LSTM cells and network architectures. Neural Comput.

[40] Xu Z, Wang S, Zhu F, Huang J (2017) Seq2seq fingerprint: An unsupervised deep molecular embedding for drug discovery. In: Proceedings of the 8th ACM International Conference on Bioinformatics, Computational Biology,and Health Informatics. ACM-BCB ’17, pp. 285–294. Association for Computing Machinery, New York, NY, USA. 10.1145/3107411.3107424

[41] Li X, Fourches D (2020) Inductive transfer learning for molecular activity prediction: Next-gen qsar models with molpmofit. J Cheminformat. 10.1186/s13321-020-00430-x 10.1186/s13321-020-00430-x PMC7178569 33430978

[42] Refaeilzadeh P, Tang L, Liu H (2009) In: Liu L, ÖZSU MT (eds) Cross-Validation, pp. 532–538. Springer, Boston, MA

[43] Wishart D, Knox C, Guo A, Cheng D, Shrivastava S, Tzur D, Gautam B, Hassanali M (2008). Drugbank: a knowledgebase for drugs, drug actions and drug targets. Nucleic Acids Res.

[44] Guo C, Pleiss G, Sun Y, Weinberger KQ (2017) On calibration of modern neural networks. In: Proceedings of the 34th International Conference on Machine Learning-Volume 70, pp. 1321–1330. JMLR. org

[45] Wieslander H, Harrison PJ, Skogberg G, Jackson S, Fridén M, Karlsson J, Spjuth O, Wählby C (2021). Deep learning with conformal prediction for hierarchical analysis of large-scale whole-slide tissue images. IEEE J Biomed Health Inform.

[46] Merity S, Keskar NS, Socher R (2017) Regularizing and Optimizing LSTM Language Models. arXiv. 10.48550/ARXIV.1708.02182. https://arxiv.org/abs/1708.02182

[47] Alvarsson J, Arvidsson McShane S, Norinder U, Spjuth O (2021). Predicting with confidence: using conformal prediction in drug discovery. J Pharm Sci.

