# Supplementary material for: Predicting protein network topology clusters from chemical structure using deep learning
Source: J Cheminform. 2022 Jul 15;14:47. doi: 10.1186/s13321-022-00622-7 (PMC9284831; doi:10.1186/s13321-022-00622-7)
Supplement: Supplementary file 1 — Additional file 1: Fig. S1 SMILES augmentation for the molecule aspirin. Fig. S2 Randomization test results for all the architectures used for different set of data. Fig. S3 Structures of the chemicals used for evaluating the model. Table S1 Functions predicted by the model for estrogen predicted for the second and third ranked clusters (based on the output softmax probabilities). [file 13321_2022_622_MOESM1_ESM.pdf]

SUPPLEMENT

Predicting protein network topology clusters from chemical structure using deep learning

Full list of author information is available at the end of the article

Supplemental fig. 1 SMILES augmentation for the molecule aspirin.

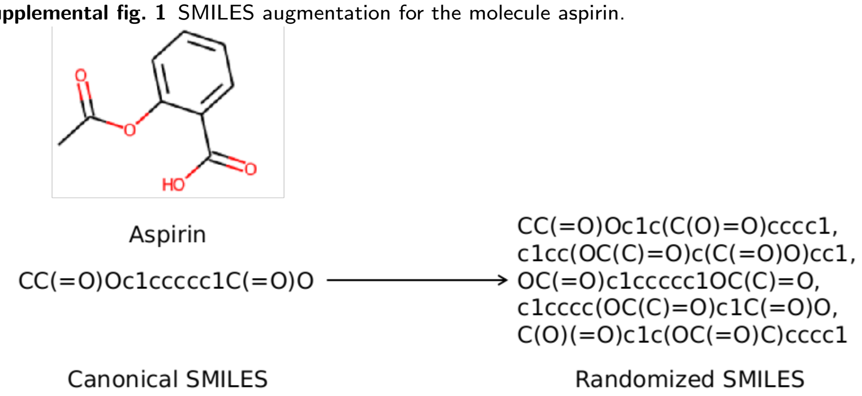

Canonical representation on the left and randomized SMILES strings on the right.

Supplemental fig. 2 Randomization test results for all the architectures used for different set of data

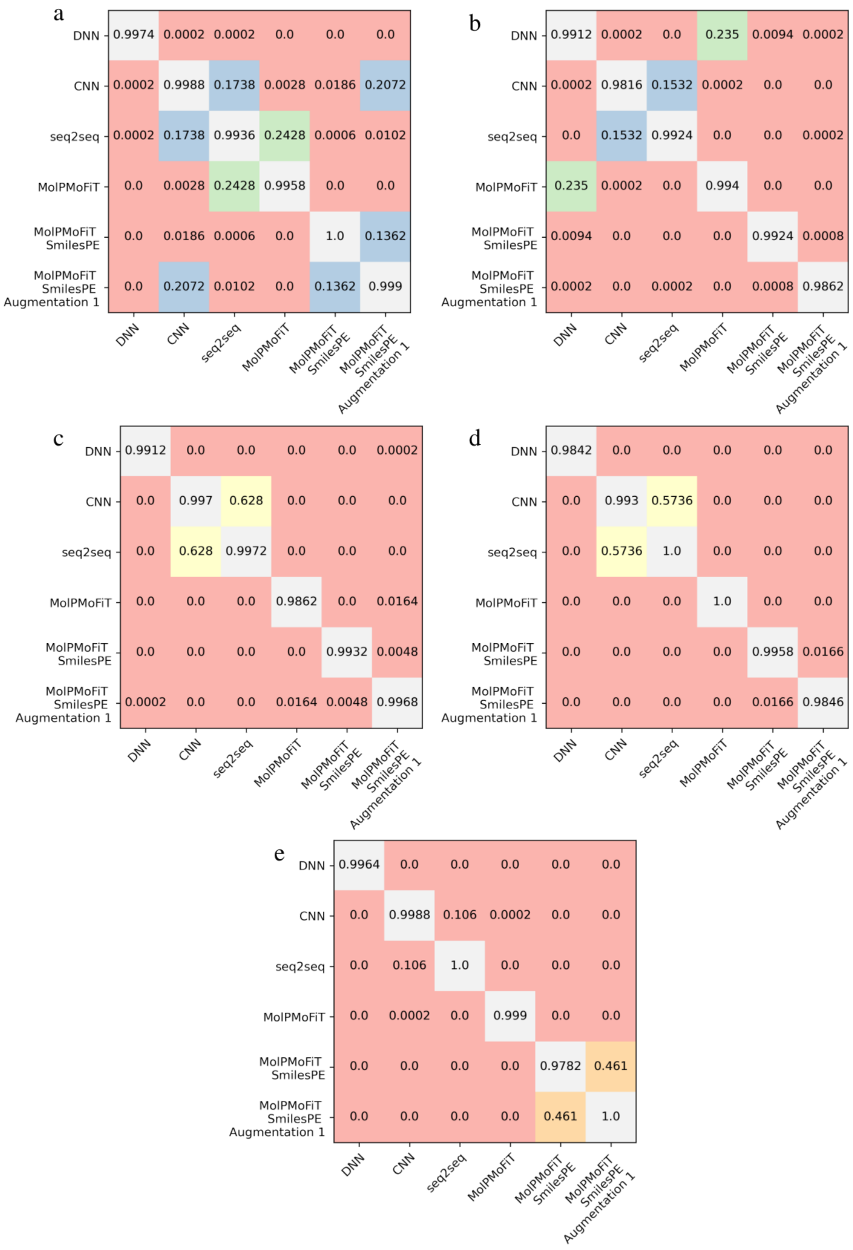

Confusion matrices for the randomization test p-values comparing the F1 scores from cross validation of the different model architectures. Results are shown for all five clustering distance thresholds explored: a) 0.001; b) 0.005; c) 0.01; d) 0.05; and e) 0.1.

Supplemental fig. 3 Structures of the chemicals used for evaluating the model

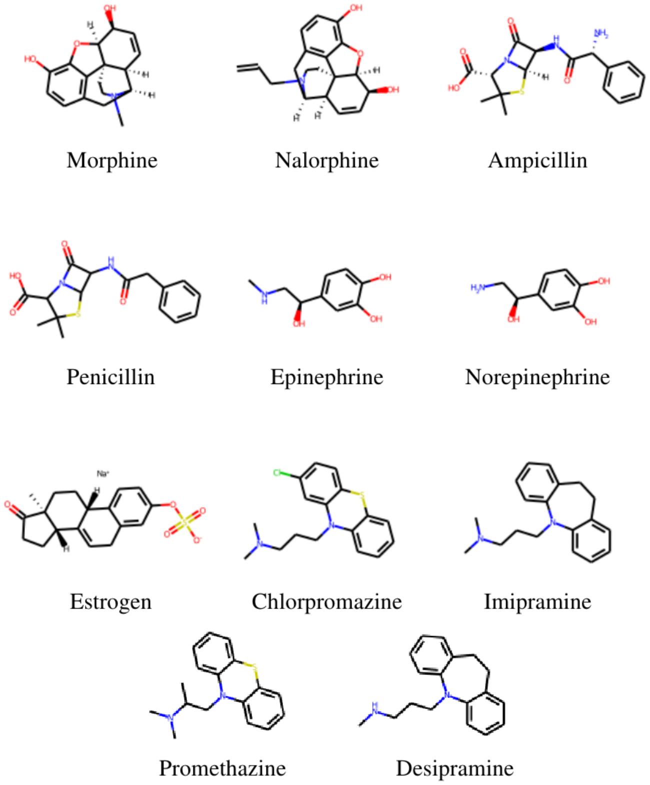

Supplemental table 1 Functions predicted by the model for estrogen predicted for the second and third ranked clusters (based on the output softmax probabilities).

Cluster rank	Qcutoff	Lcutoff	DrugBank annotation
Cluster 2	Muscarinic acetylcholine receptor M2	Muscarinic acetylcholine receptor M2, M1, M4, M3 and M5, C-C chemokine receptor type 5, Cholinesterase, Choline O-acetyltransferase	Estrogen receptor alpha and beta, Nuclear receptor subfamily 1 group I member 2, Neuronal acetylcholine receptor subunit alpha-4, G-protein coupled estrogen receptor 1, ATP synthase subunit a, Beclin-1
Cluster 3	Aromatase; Catalyzes the formation of aromatic C18 estrogens from C19 androgens	Aromatase, Steryl-sulfatase; Conversion of sulfated steroid precursors to estrogens during pregnancy, 2,4-dienoyl-CoA reductase, mitochondrial, Cytochrome P450 3A5, Phosphatidate cytidylyltransferase 2	
